# Deep-learning model for screening sepsis using electrocardiography

**DOI:** 10.1186/s13049-021-00953-8

**Published:** 2021-10-03

**Authors:** Joon-myoung Kwon, Ye Rang Lee, Min-Seung Jung, Yoon-Ji Lee, Yong-Yeon Jo, Da-Young Kang, Soo Youn Lee, Yong-Hyeon Cho, Jae-Hyun Shin, Jang-Hyeon Ban, Kyung-Hee Kim

**Affiliations:** 1 Artificial Intelligence and Big Data Research Center, Sejong Medical Research Institute, Bucheon, Republic of Korea; 2 Medical Research Team, Medical AI, Co., Seoul, Republic of Korea; 3 Department of Critical Care and Emergency Medicine, Mediplex Sejong Hospital, 20, Gyeyangmunhwa-ro, Gyeyang-gu, Incheon, Republic of Korea; 4 Medical R&D Center, Body Friend, Co., Seoul, Republic of Korea; 5 Division of Cardiology Cardiovascular Center, Mediplex Sejong Hospital, Incheon, Republic of Korea

**Keywords:** Sepsis, Shock, Septic, Infections, Electrocardiography, Deep learning, Artificial intelligence

## Abstract

**Background:**

Sepsis is a life-threatening organ dysfunction and a major healthcare burden worldwide. Although sepsis is a medical emergency that requires immediate management, screening for the occurrence of sepsis is difficult. Herein, we propose a deep learning-based model (DLM) for screening sepsis using electrocardiography (ECG).

**Methods:**

This retrospective cohort study included 46,017 patients who were admitted to two hospitals. A total of 1,548 and 639 patients had sepsis and septic shock, respectively. The DLM was developed using 73,727 ECGs from 18,142 patients, and internal validation was conducted using 7774 ECGs from 7,774 patients. Furthermore, we conducted an external validation with 20,101 ECGs from 20,101 patients from another hospital to verify the applicability of the DLM across centers.

**Results:**

During the internal and external validations, the area under the receiver operating characteristic curve (AUC) of the DLM using 12-lead ECG was 0.901 (95% confidence interval, 0.882–0.920) and 0.863 (0.846–0.879), respectively, for screening sepsis and 0.906 (95% confidence interval (CI), 0.877–0.936) and 0.899 (95% CI, 0.872–0.925), respectively, for detecting septic shock. The AUC of the DLM for detecting sepsis using 6-lead and single-lead ECGs was 0.845–0.882. A sensitivity map revealed that the QRS complex and T waves were associated with sepsis. Subgroup analysis was conducted using ECGs from 4,609 patients who were admitted with an infectious disease, and the AUC of the DLM for predicting in-hospital mortality was 0.817 (0.793–0.840). There was a significant difference in the prediction score of DLM using ECG according to the presence of infection in the validation dataset (0.277 vs. 0.574, *p* < 0.001), including severe acute respiratory syndrome coronavirus 2 (0.260 vs. 0.725, *p* = 0.018).

**Conclusions:**

The DLM delivered reasonable performance for sepsis screening using 12-, 6-, and single-lead ECGs. The results suggest that sepsis can be screened using not only conventional ECG devices but also diverse life-type ECG machines employing the DLM, thereby preventing irreversible disease progression and mortality.

## Introduction

Sepsis is a life-threatening organ dysfunction caused by dysregulation of the host response to infection and is a major healthcare problem worldwide [1, 2]. In 2017, a total of 48.9 million cases of sepsis were recorded worldwide, and 11.0 million sepsis-related deaths were reported, representing 19.7% of all the global deaths [2, 3]. Although the mortality rate associated with sepsis has decreased by 52.8% over the past 20 years, the incidence has increased, likely reflecting the aging population with more comorbidities [3]. Because sepsis is a medical emergency that requires immediate treatment and resuscitation, early recognition is a cornerstone for preventing disease progression and death [2].

Vital signs and blood tests are required to screen and diagnose sepsis [1]. Vital signs are measured by the medical staff at intervals, and blood tests require infrastructure for blood sampling and analysis. Therefore, it is difficult to monitor the occurrence of sepsis in real time in hospitals. Sepsis has its highest burden in areas with a lower sociodemographic index as these areas lack medical resources for screening, diagnosis, and treatment of sepsis [4]. Furthermore, home monitoring for the deterioration of infected patients and screening for sepsis are critical for appropriate allocation of scarce medical resources in a pandemic such as the ongoing severe acute respiratory syndrome coronavirus 2 (SARS-CoV-2) pandemic. However, the existing method for the screening of sepsis using vital signs and laboratory examinations is limited in daily living situations and remote monitoring.

A low-cost and widely available method for screening patients with sepsis has important therapeutic implications. Electrocardiography (ECG) is a noninvasive test that can be monitored in real time, and diverse wearable and life-type devices have been developed for remote monitoring and transfer. In the SARS-CoV-2 pandemic, an ECG monitoring device was used to monitor patients [5]. In previous studies, approximately 50% of patients who were diagnosed with sepsis exhibited signs of myocardial dysfunction; furthermore, a prolonged duration and decreased amplitude of the QRS complex have been reported in sepsis patients [6–9]. Artificial intelligence (AI) technologies based on deep learning have been used in diverse medical domains, and a DLM has been applied for the diagnosis of heart failure, pulmonary hypertension, valvular heart disease, electrolyte imbalance, and anemia using ECG [10–14]. In contrast to conventional statistical methods, a DLM can diagnose or predict diseases by extracting possible implications from data and capturing nonlinear and subtle changes in an ECG [15]. In this study, we developed and validated a DLM for sepsis screening using ECG. And we confirmed the performance when using 12-, 6-, and single-lead ECGs to confirm the possibility of predicting sepsis in diverse ECG devices.

## Methods

### Study design and population

**Fig. 1:**
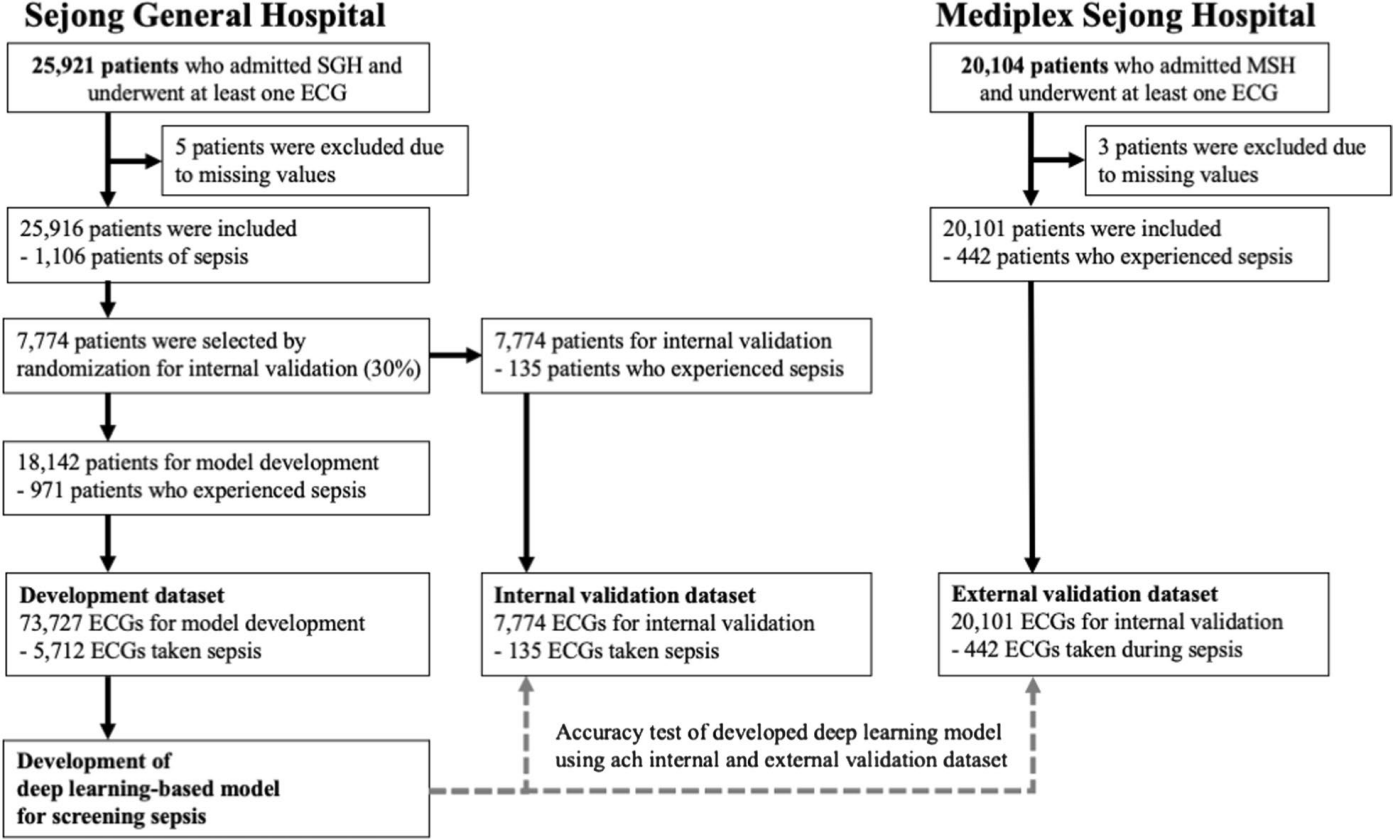
Study flowchart

We conducted a retrospective multicenter cohort study in two hospitals. The study population included adult patients who were admitted to two hospitals and underwent at least one standard 10-s 12-lead ECG during the study period. We excluded individuals with missing ECG data. Data from the Sejong General Hospital (SGH) were used to develop and validate the DLM. The patients admitted to SGH during the study period (October 2016 to November 2020) were randomly split into development (70%) and internal-validation (30%) datasets (Fig. 1). Data from the Mediplex Sejong Hospital (MSH) during the study period (March 2017 to November 2020) were only used for external validation, confirming that the developed DLM was robust across different hospitals. There were no patients that had undergone treatment both at the SGH and MSH. The patients from the two hospitals were exclusively divided. As the purpose of the validation dataset was to assess the accuracy of the DLM, we used only one ECG from each patient for the internal and external validation datasets, the time closest to the sepsis time, which was confirmed by critical care medicine physicians.

This study was approved by the Institutional Review Board (IRB) of SGH (2020–0541) and MSH (2020–149). Clinical data, including ECG, age, sex, admission note, vital signs, and laboratory examination results, were extracted from the electronic health records of both hospitals after anonymization. The IRBs of both hospitals waived the need for informed consent because this was a retrospective study using fully anonymized data, and thus, the possibility of harm to patients was unlikely.

### Predictor variable

ECG was the only predictor variable. Digitally stored 12-lead ECG data had 500 data points per second (500 Hz) at each lead for 10 s. In other words, one ECG dataset has 60,000 values. We preprocessed the ECGs for sampling, normalization, and noise filtering. Because there were more artifacts at the beginning and end of the ECG, we removed 1 s of data at the beginning and end of the ECG. And we normalized (z-score) based on the mean and standard deviation. We conducted noise filtering for decreased artifact in ECG data and used band-pass filter for noise reduction. We also normalized the value of age and changed the value of sex to one-hot encoding. We also used augmentation, the addition of linear and nonlinear noise causing baseline changes was performed. We used 8-s data of each lead. We created a dataset using 12-, 6-, and single-lead ECG datasets. We created a 12-lead ECG dataset using 12-lead ECG data (12 × 4000). We also created 6- and single-lead ECG datasets from the partial datasets of the 12-lead ECG. The 6-lead ECG dataset was created from limb 6-lead (I, II, III, aVL, aVR, aVF) and a single-lead ECG dataset was created from lead I. We selected these leads because they can be measured using diverse wearable and life-type ECG devices.

### Endpoints

The primary endpoint of this study was the presence of sepsis. Sepsis was defined as per the Third International Consensus Definitions for Sepsis and Septic Shock (Sepsis-3). Three critical care medicine physicians reviewed the medical records of the study population, including admission notes, laboratory examination results, vital signs, drug administration data, and rapid response team’s progression note, to label the presence and time range of sepsis. Septic shock was the secondary endpoint and defined based on Sepsis-3.

For the primary endpoint—sepsis in patients with suspected infectious disease—we labeled the ECG within and outside the time range of sepsis as *sepsis* and *non-sepsis*, respectively. Further, in patients who had no history of infectious diseases during hospitalization, we labeled all ECGs as *non-sepsis*. Similarly, for the secondary endpoint, namely septic shock, we labeled the ECG within the time range of septic shock as *septic shock* and the other ECG as *non-septic shock*.

### Development of DLM for detecting sepsis using ECG

**Fig. 2:**
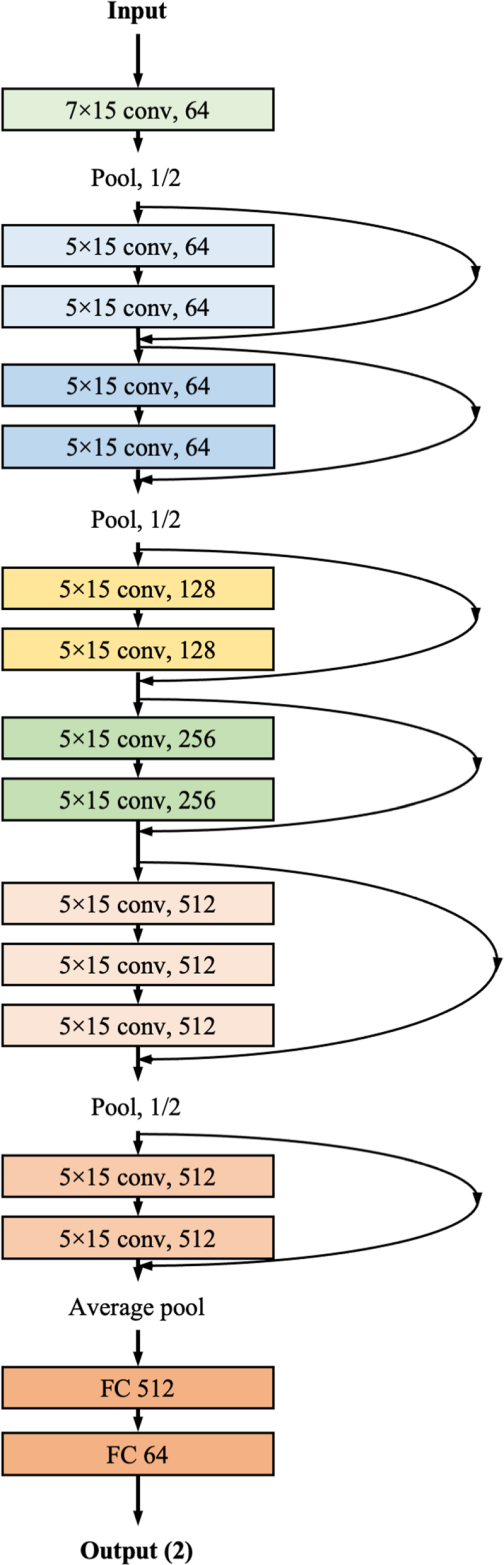
Architecture of DLM to screen sepsis using ECG. *conv* convolutional neural network layer; *DLM* deep-learning model; *ECG* electrocardiography; *FC* fully connected layer

We developed a DLM based on a convolutional neural network (residual neural networks in particular) (Fig. 2). The residual neural network contained a skip connection to avoid the problem of vanishing gradients. In a residual block with four stages, two convolutional layers and two batch normalization layers were repeated. Furthermore, there were two flattened layers in the DLM. The last layer of the seventh residual block was connected to a flattened layer that is fully connected to a one-dimensional (1D) layer composed of neural nodes. The second fully connected 1D layer was connected to the output node, which was composed of two nodes. The value of the output nodes of the DLM represents the probability of endpoints. The output node of the DLM uses a softmax function as an activation function.

### Statistical analysis

At each input ECG of the validation data, the DLM calculated the probability of sepsis within the range of zero (non-sepsis) to 1 (sepsis). To confirm the accuracy of the DLM, we compared the probability calculated by the DLM with the presence of sepsis (ground truth) in the internal and external validation datasets. Thus, we used the area under the receiver operating characteristic curve (AUC), sensitivity, specificity, positive predictive value (PPV), and negative predictive value (NPV). We confirmed the cut-off point from Youden’s J statistics in the development dataset. We then applied the cut-off point to validate the performance of internal and external validations [16]. As a comparative measure, we used C-reactive protein (CRP) and body temperature abnormality (difference between measured body temperature and 36.5 °C) to screen for sepsis and septic shock.

Continuous variables were presented as mean values (standard deviation, SD) and compared using the unpaired Student’s *t*-test or Mann–Whitney U test. Categorical variables were expressed as frequencies and percentages and were compared using the χ2 test. The exact 95% CIs were used for all measures of diagnostic performance, except for AUC. The CIs for the AUC were determined based on the Sun and Su optimization of the De-long method using the pROC package in R (R Foundation for Statistical Computing, Vienna, Austria). A significant difference in patient characteristics was defined as a two-sided *p value* < 0.05. Statistical analyses were performed using the R software, version 3.4. In addition, PyTorch’s open-source software library was used for the backend and Python (version 3.6) for the analysis[17].

### Visualizing the developed DLM for interpretation

To compare the findings from the developed DLM with the current medical knowledge, we used a sensitivity map using the saliency method [18, 19]. The map shows the region with a significant effect on the decision of the DLM. The sensitivity map was computed based on the first-order gradients of the classifier probabilities with respect to the input signals; if the probability of a classifier is sensitive to a specific region of the signal, the region would be important in the decision of the DLM. Using this method, we verified that the region of the ECG was correlated with sepsis. We used a gradient class activation map (Grad-CAM) as the sensitivity map. We could not find a definite decision process for the developed deep-learning model. Instead, we calculated the importance of the selected variables. We also confirmed the variable importance of the ECG features in the conventional statistical method (logistic regression) and machine-learning methods (random forest and deep learning). We calculated the variable importance of logistic regression, random forest, and deep learning based on the difference in deviance, mean degreased Gini, and Garson’s relative importance, respectively.

### Verifying DLM performance to predict in-hospital mortality among infectious disease patients

We hypothesized that the ECGs could display severity in infectious diseases and that the developed DLM would predict in-hospital mortality of patients with infectious diseases. In other words, we hypothesized that a high DLM score is correlated with a severe infectious disease. We conducted a subgroup analysis of patients with suspected infectious diseases in the internal and external validation datasets. We verified the in-hospital mortality prediction performance of the DLM with these patients. To confirm the accuracy of the DLM, we compared the score calculated by the DLM with the presence of in-hospital mortality in the subgroup datasets. For comparison, we used the sequential organ failure assessment (SOFA) score, quick SOFA score, National Early Warning Score (NEWS), Modified Early Warning Score (MEWS), lactate, white blood cell (WBC) count, and CRP to predict in-hospital mortality among infectious disease patients [20–23].

## Results

The eligible study population included patients admitted to the SGH and MSH. As shown in Fig. 1, we excluded eight patients because of missing clinical information including that of ECGs, admission notes, and laboratory examination results. The study involved 46,017 patients, of which 1,548 and 639 patients had sepsis and septic shock, respectively. The DLM was developed using a development dataset of 73,727 ECGs from 18,142 patients from the SGH. The internal validation of the DLM performance was conducted using 7,774 ECGs from 7,774 patients from the SGH. External validation of the DLM was conducted using 20,101 ECGs from 20,101 MSH patients. The patients were divided into development, internal validation, and external validation groups. In patients with sepsis, the ECG had a rightward P-, R-, and T-wave axes, prolonged QTc, and tachycardia (Table 1).

**Table 1:** Baseline characteristics

Characteristics	Non-sepsis patients (n = 44,469)	Sepsis patients (n = 1,548)	*p*
Age, year, mean (SD)	58.01 (19.83)	61.83 (24.93)	< 0.001
Male, n (%)	20,836 (46.9)	810 (52.3)	< 0.001
Systolic blood pressure, mmHg, mean (SD)	121.61 (33.43)	101.05 (39.53)	< 0.001
Heart rate, bpm, mean (SD)	76.92 (17.63)	103.59 (23.65)	< 0.001
Respiratory rate, bpm, mean (SD)	18.79 (4.34)	26.29 (8.72)	< 0.001
Peripheral oxygen saturation, %, mean (SD)	97.18 (16.97)	92.81 (28.20)	< 0.001
Mental change, n (%)	288 (0.6)	753 (48.6)	< 0.001
C-reactive protein, mg/dL, mean (SD)	13.30 (37.22)	49.80 (74.46)	< 0.001
Lactate, mmol/L, mean (SD)	1.87 (1.87)	4.64 (5.03)	< 0.001
White blood cell count, per µL, mean (SD)	8180 (4200)	13,090 (6190)	< 0.001
Total bilirubin, mg/dL, mean (SD)	0.72 (0.83)	1.39 (2.58)	< 0.001
Creatinine, mg/dL, mean (SD)	0.98 (0.97)	1.47 (1.53)	< 0.001
PR interval, ms, mean (SD)	169.07 (31.53)	161.47 (41.24)	< 0.001
QRS duration, ms, mean (SD)	96.59 (18.91)	96.96 (23.49)	0.461
QT interval, ms, mean (SD)	401.65 (47.90)	372.11 (63.62)	< 0.001
QTc, ms, mean (SD)	442.41 (37.66)	469.16 (43.79)	< 0.001
P-wave axis, degree, mean (SD)	43.42 (30.85)	45.50 (38.72)	0.033
R-wave axis, degree, mean (SD)	38.40 (46.36)	47.11 (61.76)	< 0.001
T-wave axis, degree, mean (SD)	47.42 (53.10)	66.09 (81.47)	< 0.001

*SD* standard deviation

**Fig. 3:**
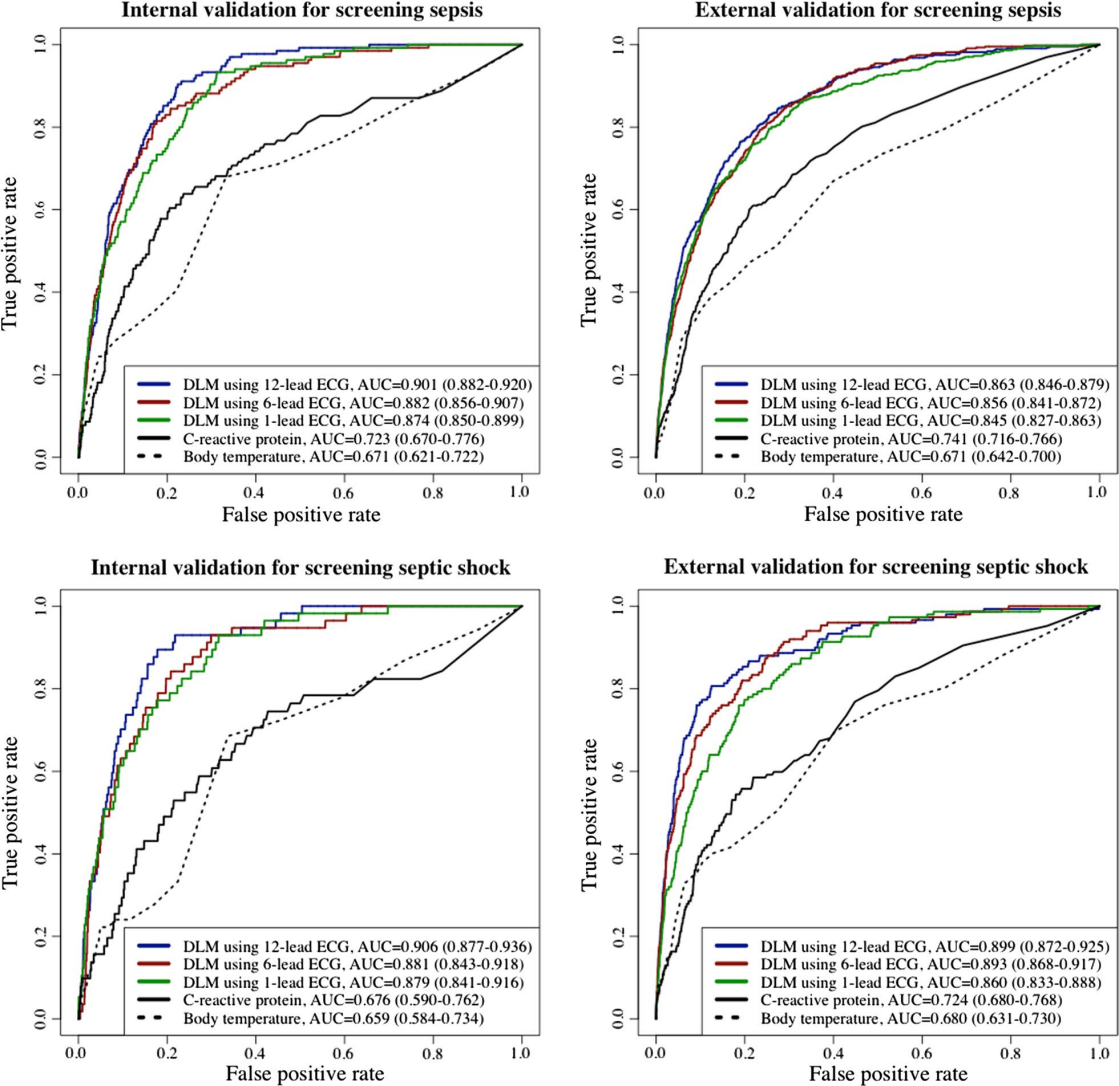
Performance of DLM for screening sepsis and septic shock using electrocardiography. *AUC* area under the receiver operating characteristic curve; *ECG* electrocardiography; *NPV* negative predictive value; *PPV* positive predictive value; *SEN* sensitivity; *SPE* specificity

During the internal and external validations, the AUC of the DLM for detecting sepsis, the primary outcome, using a 12-lead ECG was 0.901 (95% CI = 0.882–0.920) and 0.863 (95% CI = 0.846–0.879), respectively (Fig. 3 and Table 2). The AUC of the DLM for detecting septic shock using 12-lead ECGs during internal and external validations was 0.906 (95% CI = 0.877–0.936) and 0.899 (95% CI = 0.872–0.925), respectively. The AUC of the DLM for detecting sepsis using 6-lead and single-lead ECGs was 0.845–0.882, and the AUC of the DLM for detecting septic shock using 6-lead and single-lead ECGs was 0.881–0.906.

**Table 2:** Performance of DLM for screening sepsis and septic shock using electrocardiography

Prediction model	Internal validation					External validation				
	AUC (95% CI)	SEN (95% CI)	SPE (95% CI)	PPV (95% CI)	NPV (95% CI)	AUC (95% CI)	SEN (95% CI)	SPE (95% CI)	PPV (95% CI)	NPV (95% CI)
*Performance for screening sepsis*										
DLM using 12-lead ECG	0.901 (0.882–0.920)	0.904 (0.854–0.953)	0.776 (0.767–0.785)	0.067 (0.055–0.078)	0.998 (0.997–0.999)	0.863 (0.846–0.879)	0.765 (0.725–0.804)	0.810 (0.805–0.816)	0.083 (0.075–0.091)	0.994 (0.992–0.995)
DLM using 6-lead ECG	0.882 (0.856–0.907)	0.815 (0.749–0.880)	0.824 (0.816–0.833)	0.076 (0.062–0.089)	0.996 (0.994–0.998)	0.856 (0.841–0.872)	0.794 (0.756–0.832)	0.766 (0.760–0.772)	0.071 (0.064–0.078)	0.994 (0.993–0.995)
DLM using 1-lead ECG	0.874 (0.850–0.899)	0.933 (0.891–0.975)	0.688 (0.678–0.698)	0.050 (0.042–0.059)	0.998 (0.997–0.999)	0.845 (0.827–0.863)	0.796 (0.759–0.834)	0.746 (0.739–0.752)	0.066 (0.059–0.072)	0.994 (0.993–0.995)
C-reactive protein	0.723 (0.670–0.776)	0.638 (0.550–0.725)	0.763 (0.748–0.778)	0.091 (0.071–0.111)	0.983 (0.977–0.988)	0.741 (0.716–0.766)	0.607 (0.561–0.654)	0.783 (0.776–0.791)	0.087 (0.077–0.097)	0.983 (0.981–0.986)
Body temperature	0.671 (0.621–0.722)	0.679 (0.599–0.759)	0.667 (0.655–0.679)	0.041 (0.032–0.049)	0.990 (0.987–0.993)	0.671 (0.642–0.700)	0.669 (0.624–0.714)	0.601 (0.593–0.608)	0.044 (0.039–0.049)	0.985 (0.983–0.987)
*Performance for screening septic shock*										
DLM using 12-lead ECG	0.906 (0.877–0.936)	0.895 (0.815–0.974)	0.822 (0.814–0.831)	0.036 (0.026–0.045)	0.999 (0.998–1.000)	0.899 (0.872–0.925)	0.807 (0.743–0.870)	0.875 (0.871–0.880)	0.046 (0.038–0.054)	0.998 (0.998–0.999)
DLM using 6-lead ECG	0.881 (0.843–0.918)	0.842 (0.747–0.937)	0.792 (0.783–0.801)	0.029 (0.021–0.037)	0.999 (0.998–0.999)	0.893 (0.868–0.917)	0.880 (0.828–0.932)	0.749 (0.743–0.755)	0.026 (0.021–0.030)	0.999 (0.998–0.999)
DLM using 1-lead ECG	0.879 (0.841–0.916)	0.930 (0.864–0.996)	0.684 (0.673–0.694)	0.021 (0.016–0.027)	0.999 (0.999–1.000)	0.860 (0.833–0.888)	0.773 (0.706–0.840)	0.801 (0.795–0.806)	0.028 (0.023–0.033)	0.998 (0.997–0.999)
C-reactive protein	0.676 (0.590–0.762)	0.745 (0.625–0.865)	0.573 (0.556–0.590)	0.027 (0.019–0.036)	0.993 (0.989–0.997)	0.724 (0.680–0.768)	0.585 (0.505–0.665)	0.781 (0.774–0.788)	0.030 (0.023–0.036)	0.994 (0.992–0.995)
Body temperature	0.659 (0.584–0.734)	0.685 (0.561–0.809)	0.663 (0.651–0.674)	0.017 (0.011–0.022)	0.996 (0.994–0.998)	0.680 (0.631–0.730)	0.697 (0.622–0.773)	0.596 (0.588–0.604)	0.016 (0.013–0.019)	0.995 (0.994–0.997)

*AUC* area under the receiver operating characteristic curve; *DLM* deep learning-based model; *ECG* electrocardiography; *NPV* negative predictive value; *PPV* positive predictive value; *SEN* sensitivity; *SPE* specificity

**Fig. 4:**
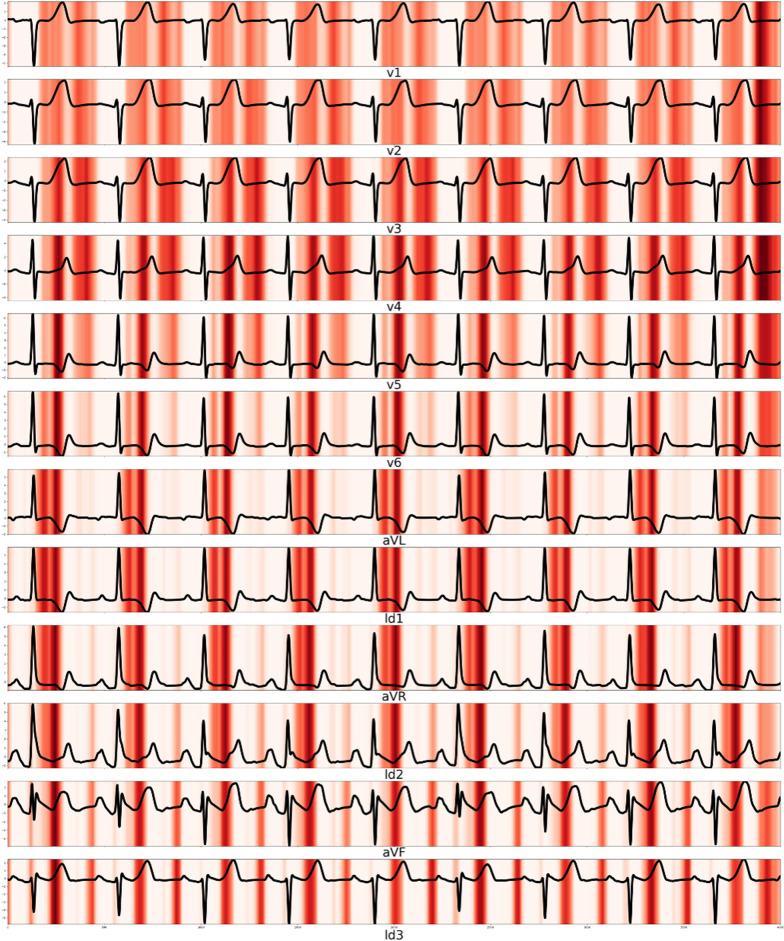
Sensitivity map of septic shock patients

A sensitivity map showed that the QT interval and T wave were associated with sepsis, and the variable importance of deep learning confirmed that prolonged QTc was associated with sepsis (Fig. 4). The logistic regression and random forest had different variable importance and showed that prolonged QTc, T axis, and QRS duration were important variables (Table 3).

**Table 3:** Variable importance for detecting sepsis

Rank	Logistic regression (deviance difference)	Random forest (mean decrease Gini)	Deep learning (relative importance)
1	QTc (492)	Heart rate (473.2)	QTc (0.193)
2	Age (274)	T-wave axis (472.1)	QT interval (0.168)
3	QRS duration (207)	R-wave axis (443.2)	PR interval (0.121)
4	T-wave axis (145	QTc (429.5)	T-wave axis (0.085)
5	QT interval (101)	P-wave axis (413.3)	QRS duration (0.082)
6	Heart rate (63)	Age (394.5)	Age (0.079)
7	P-wave axis (18)	QRS duration (386.2)	Heart rate (0.078)
8	R-wave axis (11)	QT interval (367.0)	P-wave axis (0.075)
9	PR interval (2)	PR interval (363.0)	R-wave axis (0.063)
10	Sex (− 1)	Sex (0.1)	Sex (0.055)

**Fig. 5:**
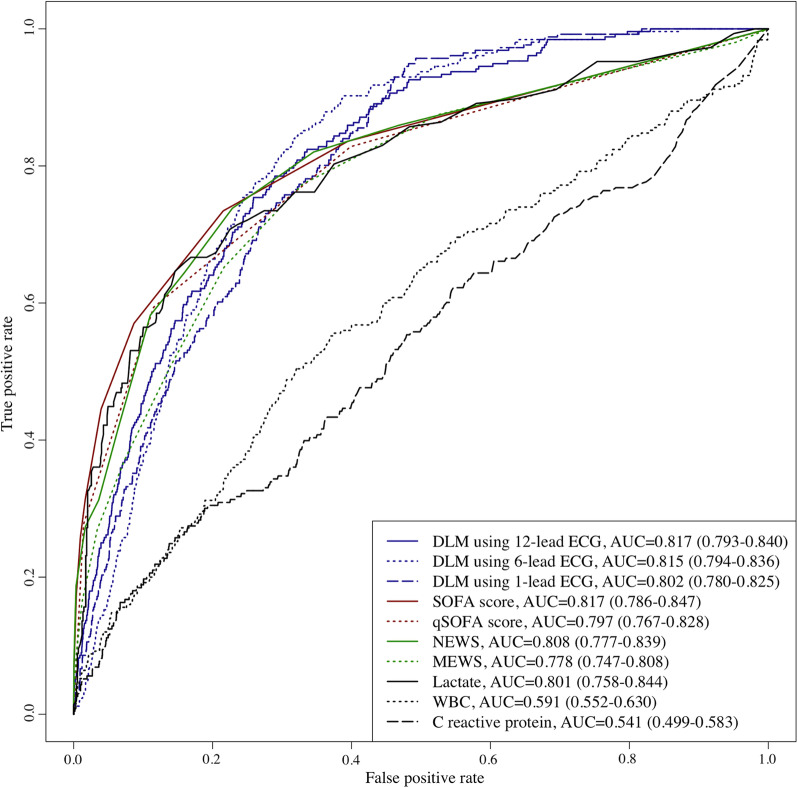
Performance of DLM for predicting in-hospital mortality of patients with infectious disease. *AUC* area under the receiver operating characteristic curve; *ECG* electrocardiography; *MEWS* Modified Early Warning Score; *NEWS* National Early Warning Score; *NPV* negative predictive value; *PPV* positive predictive value; *SEN* sensitivity; *SOFA* sequential organ failure assessment; *SPE* specificity

Subgroup analysis was conducted using ECGs from 4,609 patients who were grouped into the validation dataset with infectious diseases. There were 256 in-hospital mortality cases in the subgroup study population. The AUC of the DLM using 12-, 6-, and single-lead ECG, SOFA, qSOFA, NEWS, MEWS, lactate, WBC, and CRP for predicting in-hospital mortality was 0.817 (0.793–0.840), 0.815 (0.794–0.836), 0.802 (0.780–0.825), 0.817 (0.786–0.847), 0.797 (0.767–0.828), 0.808 (0.777–0.839), 0.778 (0.747–0.808), 0.801 (0.758–0.844), 0.591 (0.552–0.630), and 0.541 (0.499–0.583), respectively, which outperformed other predictive models (Fig. 5 and Table 4).

**Table 4:** Performance of DLM for predicting in-hospital mortality of patients with infectious diseases

Predictive model	AUC (95% CI)	SEN (95% CI)	SPE (95% CI)	PPV (95% CI)	NPV (95% CI)
DLM using 12-lead ECG	0.817 (0.793–0.840)	0.785 (0.735–0.835)	0.710 (0.697–0.724)	0.137 (0.120–0.155)	0.983 (0.978–0.987)
DLM using 6-lead ECG	0.815 (0.794–0.836)	0.844 (0.799–0.888)	0.682 (0.668–0.695)	0.135 (0.118–0.152)	0.987 (0.983–0.991)
DLM using 1-lead ECG	0.802 (0.780–0.825)	0.945 (0.917–0.973)	0.524 (0.509–0.539)	0.105 (0.092–0.117)	0.994 (0.991–0.997)
SOFA score	0.817 (0.786–0.847)	0.734 (0.680–0.788)	0.785 (0.773–0.797)	0.167 (0.145–0.189)	0.980 (0.976–0.985)
Quick SOFA score	0.797 (0.767–0.828)	0.594 (0.534–0.654)	0.885 (0.875–0.894)	0.233 (0.200–0.265)	0.974 (0.969–0.979)
NEWS	0.808 (0.777–0.839)	0.738 (0.684–0.792)	0.771 (0.759–0.784)	0.160 (0.139–0.180)	0.980 (0.976–0.985)
MEWS	0.778 (0.747–0.808)	0.773 (0.722–0.825)	0.670 (0.656–0.684)	0.121 (0.105–0.137)	0.981 (0.976–0.985)
Lactate	0.801 (0.758–0.844)	0.646 (0.569–0.724)	0.854 (0.829–0.879)	0.466 (0.397–0.534)	0.925 (0.905–0.944)
WBC	0.591 (0.552–0.630)	0.504 (0.442–0.566)	0.679 (0.665–0.694)	0.086 (0.072–0.101)	0.958 (0.951–0.965)
C-reactive protein	0.541 (0.499–0.583)	0.300 (0.242–0.359)	0.811 (0.799–0.823)	0.084 (0.065–0.103)	0.953 (0.946–0.960)

*AUC* area under the receiver operating characteristic curve; *ECG* electrocardiography; *MEWS* Modified Early Warning Score; *NEWS* National Early Warning Score; *NPV* negative predictive value; *PPV* positive predictive value; *SEN* sensitivity; *SOFA* sequential organ failure assessment; *SPE* specificity

**Fig. 6:**
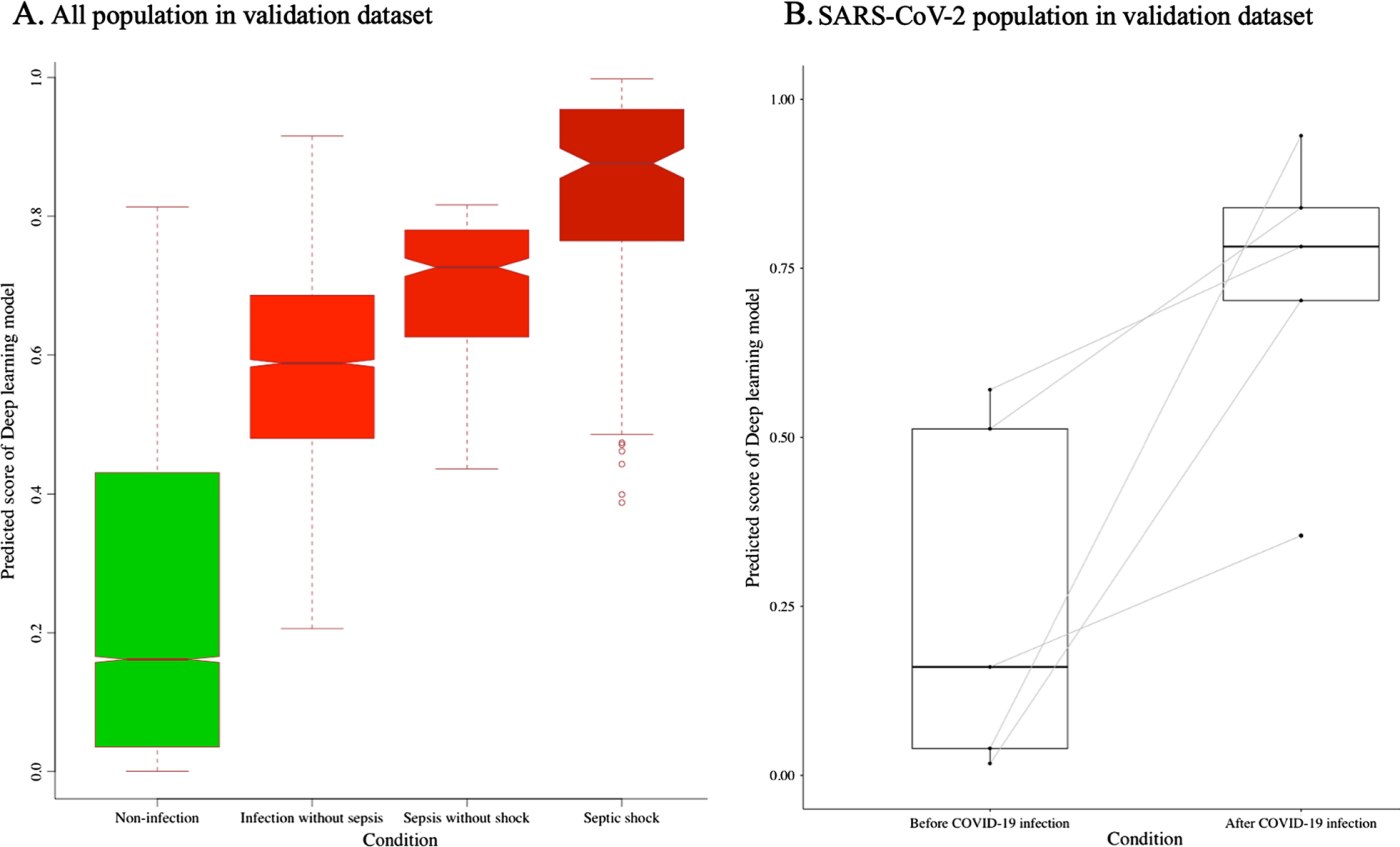
Change of DLM’s prediction score according to infection. *SARS-CoV-2* Severe acute respiratory syndrome coronavirus 2

As shown in Fig. 6, there was a significant difference in the prediction score of the DLM using ECG according to the presence of infection in the validation dataset (0.277 vs. 0.574, *p* < 0.001). In patients with SARS-CoV-2, the same trend was observed in the prediction score of DLM using ECG before and after SARS-CoV-2 infection (0.260 vs. 0.725, *p* = 0.018).

## Discussion

We developed a DLM for screening sepsis and septic shock using 12-, 6-, and single-lead ECGs and demonstrated reasonable accuracies for internal and external validations. We confirmed the performance of predicting in-hospital mortality in a subgroup analysis of patients with infectious diseases. We also identified the ECG regions and features associated with sepsis. To the best of our knowledge, this study is the first to develop a DLM for sepsis screening using ECG.

Approximately 50% of sepsis patients have cardiac dysfunction, which is a well-known risk factor associated with a significantly increased mortality rate of 20–50% [24]. Sepsis develops into cardiac dysfunction by decreasing the beta-adrenergic receptor components, which are mediated by inflammatory substances such as cytokines and nitric oxide [25]. Direct cardiomyocyte injury or death is caused by toxins and complications from sepsis. Cardiomyocyte apoptosis is the leading cause of cardiac dysfunction, followed by the downregulation of beta-adrenoreceptors and impairment of myofibril function owing to the disruption of calcium liberation. Because sepsis affects cardiac function through direct or indirect pathophysiology, we hypothesized that an ECG contains information for sepsis detection. Previously, Rich et al. showed that the QRS amplitude of sepsis was smaller than that of normal individuals [9]. However, conventional statistical methods, such as logistic regression, cannot develop diagnostic criteria for using these subtle changes and nonlinear correlations. ECG is affected by not only cardiac function but also other human factors. For example, a patient with fat and a larger body mass index has a lower ECG amplitude [26]. Madias et al. reported that the loss of QRS amplitude in the ECGs in patients with sepsis is not due to cardiac dysfunction but due to an extracardiac reason such as a reduction in the transfer impedance of the body volume conductor owing to water accumulation [27]. Recent studies have highlighted the possibility of using AI for interpreting an ECG. Using AI technologies based on a DLM, we could diagnose diseases that could not be diagnosed based on previous medical knowledge such as heart failure, valvular heart disease, pulmonary hypertension, anemia, and hyperkalemia [11–14, 28–30]. The most important aspect of deep learning is its ability to extract features and develop an algorithm using various types of data such as images, 2D data, and waveforms [15]. In this study, we developed a DLM for detecting sepsis and validated its performance based on external validation. DLM can also detect septic shock using a DLM prediction score. Previous studies have shown that inflammatory markers and infection are closely correlated with cardiac disease and ECG [31].

There has been enormous development in diverse wearable and lifestyle devices worldwide. There is already a base for remote diagnosis and treatment based on diverse biosensors and internet technologies. However, there are limitations in the biosignal interpretation by various wearable devices. ECG is an important biosignal for remote medical monitoring and treatment as it can be measured using diverse wearable devices and transferred to remote medical sites in real time. As a conventional statistical limitation, an ECG is only used for the diagnosis of arrhythmia and myocardial infarction. Based on current studies, AI technologies have enabled the diagnosis and prediction of diverse diseases using ECG. In the ongoing SARS-CoV-2 pandemic, such technologies are important for screening infectious diseases, monitoring patient status, and capturing the deterioration of patients. In this study, we highlighted the possibility of using DLMs for screening infectious diseases, including SARS-CoV-2, as shown in Fig. 6. The results were not definite evidence of SARS-CoV-2 screening via ECG. However, we wanted to demonstrate the possibility of developing deep learning for SARS-CoV-2 for other researchers. There is a need for studies on the use of AI for screening sepsis and septic shock. However, this study highlights the possibility of applying ECG to detect and monitor infectious patients. In this study, we confirmed that the performance was secured in six- and single-lead ECGs. Because of this, we showed the possibility of applying the deep-learning model to various lifestyle ECG devices and patch devices.

This study had some limitations. First, we validated the DLM using retrospective data; however, it is necessary to validate the DLM using prospective studies and real-time data. Studies related to the clinical significance of this new technology are required for its application in clinical practice. In our next study, we intend to verify the DLM performance and significance through a prospective study on daily clinical practice. And we plan to conduct research on deep-learning models for predicting the development and resolution of sepsis using ECG. We plan to conduct a prospective study to validate the performance of the deep-learning model as a screening and prognostic method. Second, this study was conducted in only two hospitals in Korea, and it would be helpful to validate the DLM in patients from other countries. Third, deep learning had *a black box* limitation owing to which we could not determine the exact decision-making process. Therefore, we could not confirm that our study findings represented correlation or causality. In our next study, we intend to develop a method for confirming the decision process and the causality of the deep-learning model. For the same reason, we could not know the exact features of the ECG that were used in deep learning. As technologies for explainable deep learning that could define the reason and feature are being developed, we can use this technology in our next study. Fourth, we conducted a retrospective study, and there could be confounders in this study. A prospective randomized controlled study is needed to exclude hidden confounders and confirm the exact clinical implications of deep-learning models for sepsis.

## Conclusion

The DLM demonstrated accurate performance in detecting sepsis and septic shock using ECG. The results of the present study indicate that the application of AI technologies based on a DLM to an ECG could predict the development of sepsis in patients and enable the screening of diverse infectious diseases.

## Data Availability

The data underlying this article will be shared upon reasonable request to the corresponding author.

## Abbreviations

AUC: Area under the receiver operating characteristic curve
CI: Confidence interval
CRP: C-reactive protein
DLM: Deep learning-based model
ECG: Electrocardiography
MEWS: Modified Early Warning Score
NEWS: National Early Warning Score
NPV: Negative predictive value
PPV: Positive predictive value
SARS-CoV-2: Severe acute respiratory syndrome coronavirus 2
Sepsis-3: Third International Consensus Definitions for Sepsis and Septic Shock
SGH: Sejong General Hospital
SOFA: Sequential organ failure assessment
MSH: Mediplex Sejong Hospital
IRB: Institutional Review Board
WBC: White blood cell
1D: One-dimensional
